# Retraction: The influence of vermicomposting on photosynthetic activity and productivity of maize (*Zea mays L*.) crop under semi-arid climate

**DOI:** 10.1371/journal.pone.0272414

**Published:** 2022-08-17

**Authors:** The PLOS ONE Editors

The *PLOS ONE* Editors retract this article [1] because it was identified as one of a series of submissions for which we have concerns about authorship, competing interests, and peer review. We regret that the issues were not addressed prior to the article’s publication.

LA agreed with the retraction. MY, HZ, MWA, MNA, AMES, ATKZ, YL, and MA did not agree with the retraction. TL, WA, SH, TAH, AH, GAK, and MME either did not respond directly or could not be reached.

## References

[1] Younas M, Zou H, Laraib T, Abbas W, Akhtar MW, Aslam MN, et al. (2021) The influence of vermicomposting on photosynthetic activity and productivity of maize (*Zea mays L*.) crop under semi-arid climate. PLoS ONE 16(8): e0256450. 10.1371/journal.pone.0256450 34432836 PMC8386841

